# Combination of *Bauhinia monandra* extract and laser acupuncture reduces carrageenan-induced paw edema

**DOI:** 10.1007/s10103-026-04986-2

**Published:** 2026-08-10

**Authors:** Allyne Aparecida Dias da Silva Castro, Rogério Carvalho da Costa Junior, Eloisa Acerbi Laurindo, Lorena Campos Soares, Tatyane Matos Fernandes, Nerilson Marques Lima, Diego Mendes Xavier, Murilo Xavier Oliveira, Sandra Bertelli Ribeiro Castro, Caio César de Souza Alves, Alessandra de Paula Carli

**Affiliations:** 1 Universidade Federal dos Vales do Jequitinhonha e Mucuri, Diamantina, Brazil; 2 Federal University of Lavras, Lavras, Brazil

**Keywords:** Laser acupuncture, Photobiomodulation, *Bauhinia monandra*, Anti-inflammatory agents, Plant extracts

## Abstract

Purpose: To investigate the anti-inflammatory effect of the ethanolic extract of *Bauhinia monandra* Kurz (EEB) alone and combined with laser acupuncture (LA) in a carrageenan-induced paw edema model. Methods: EEB was characterized using HPLC–HRMS and tested for cell viability and nitric oxide (NO) production in RAW 264.7 and J774A.1 macrophages. In vivo, female BALB/c mice with paw edema were treated with EEB (100 or 200 mg/kg) alone, combined with LA (808 nm, 120 mW, 3 J, 25 s) at acupoints ST36 and SP6, or local laser. Paw edema was measured up to 4 h post-induction, and cytokine levels (IL-1$\beta$ and IL-6) were quantified. Results: EEB showed no cytotoxicity and significantly reduced NO production in vitro. Phytochemical analysis revealed a predominance of phenolic compounds, including flavonoids. In vivo, both EEB and LA significantly reduced edema. Notably, the EEB200 + LA group produced reductions in paw edema and pro-inflammatory cytokines comparable to the dexamethasone group. Conclusion: EEB demonstrated anti-inflammatory activity both in vitro and in vivo. Combining EEB (200 mg/kg) with LA yielded a more consistent anti-inflammatory response than either treatment alone, particularly in reducing edema and pro-inflammatory cytokines. These findings support further investigation, though larger sample sizes and chronic models are required before clinical claims.

**Supplementary Information:**

The online version contains supplementary material available at 10.1007/s10103-026-04986-2.

## Introduction

Although the inflammatory process is an essential biological response, its exacerbation is associated with conditions that significantly reduce quality of life [1, 2]. Conventional treatment is based on nonsteroidal anti-inflammatory drugs (NSAIDs) and corticosteroids. However, the prolonged use of these drugs leads to serious adverse systemic effects, underscoring the need for new, safer therapeutic approaches [2, 3].

Experimental models of inflammation have been widely used for the screening and assessment of the anti-inflammatory potential of new substances. The carrageenan-induced model of paw edema is one of the most common in preclinical studies, as it reproduces events characteristic of acute inflammation, such as increased vascular permeability, plasma leakage, the infiltration of inflammatory cells, and an increase in pro-inflammatory mediators [4, 5]. Among these mediators, the pro-inflammatory cytokines IL-1β and IL-6 stand out, playing a central role in both the amplification and maintenance of the inflammatory response. IL-1β promotes leukocyte activation and recruitment, contributing to increased vascular permeability, whereas IL-6 participates in acute-phase signaling and the activation of inflammatory cells. Consequently, the downregulation of these mediators is frequently utilized as an indicator of the anti-inflammatory activity of novel substances and therapeutic interventions [6, 7]. Furthermore, exacerbated production of pro-inflammatory mediators can lead to tissue damage and disease aggravation, which has driven the search for new natural compounds and therapeutic approaches capable of modulating these inflammatory pathways [6, 8]. Within this context, plant species of the genus *Bauhinia* (Fabaceae) have garnered increasing interest due to their richness in secondary metabolites and proven anti-inflammatory and antioxidant properties [9–11]. Among the species of this genus, *Bauhinia monandra* Kurz is widely used in folk medicine and has documented antioxidant, anti-inflammatory, and hypoglycemic properties [10–12]. Phytochemical studies have demonstrated that species of the genus *Bauhinia* constitute major sources of flavonoids, glycosylated flavonoids, phenolic compounds, and terpenoids—metabolites frequently associated with the modulation of inflammatory mediators and oxidative stress [13].

However, its anti-inflammatory potential remains under-explored in controlled experimental models. In addition to herbal approaches, non-pharmacological therapies have gained prominence in the control of inflammation. Laser acupuncture (LA), which is a photobiomodulation modality that delivers low-level laser irradiation to acupuncture points, has achieved anti-inflammatory, analgesic, and biostimulatory effects [14, 15]. Experimental evidence demonstrates that LA can modulate the inflammatory response by reducing pro-inflammatory cytokines, decreasing oxidative stress, and increasing mitochondrial ATP production, thus favoring tissue regeneration [14–16].

Despite the recognized benefits of phytotherapy and LA when employed separately, the investigation of the potentiated effect between these approaches remains a significant gap in the scientific literature. The combination of these treatment modalities is promising, as it unites the inhibition of inflammatory pathways by phytochemicals and cellular biostimulation promoted by laser irradiation. To explore this hypothesis and meet the demand for safer interventions, the aim of the present study was to investigate the anti-inflammatory effect of the ethanolic extract of *Bauhinia monandra Kurz* (EEB) alone and in combination with laser acupuncture in a carrageenan-induced model of paw edema to support the development of new integrative approaches. We hypothesized that the combination of EEB and LA would produce a greater anti-inflammatory effect than either treatment alone.

## Materials and methods

### Plant material and preparation of ethanolic extract of Bauhinia monandra Kurz

Leaves of *B. monandra* Kurz were collected from the municipality of Pavão in the state of Minas Gerais, Brazil (17°26’01.6"S, 40°59’50.7"W), in May 2024. A voucher specimen was deposited in the Jeanine Felfili Dendrologic Herbarium of Universidade Federal dos Vales do Jequitinhonha e Mucuri (UFVJM) (DIAM 10488) and access to the genetic heritage was registered in SisGen (AFFF12E). After washing and crushing, the fresh plant material was submitted to extraction with absolute ethanol (1:4 w/v), using 100 g of the plant for each preparation. Extraction was performed for seven days at room temperature in the absence of light with intermittent stirring. The mixture was heated then to 55 °C for 1 h, filtered, and concentrated in an evaporator at 40 °C, obtaining a final yield of 5 g of dry extract (yield: 5% w/w). The extract was frozen at − 80 °C, lyophilized, and stored at − 20 °C until use. For the in vitro assays, the lyophilized extract was reconstituted in sterile dimethyl sulfoxide (DMSO) and subsequently diluted in culture medium, obtaining a stock solution of 20 mg/mL. Subsequently, serial dilutions were performed in supplemented Roswell Park Memorial Institute (RPMI) 1640 medium. For in vivo assays, the lyophilized ethanolic extract was resuspended in phosphate buffered saline [1000 µL] and used as a vehicle. The solutions were prepared immediately before administration at the concentrations necessary to obtain doses of 100 and 200 mg/kg administered orally (gavage technique). The control group received PBS alone as the vehicle under the same experimental conditions as the treated groups.

### Phytochemical analysis by HPLC–HRMS

The chemical characterization of EEB was performed via high-performance liquid chromatography coupled to high-resolution mass spectrometry (HPLC-HRMS). An HPLC-UV 1220 Infinity II system coupled to a Q-Exactive Hybrid Quadrupole-Orbitrap spectrometer in positive electrospray mode (ESI⁺) was used, with a scanning range of *m/z* 100–1500. The structural annotation of the metabolites was based on the MS/MS fragmentation profiles, using in silico tools (MetFrag and CFM-ID) and the Global Natural Products Social Molecular Networking (GNPS) platform.

### Cell viability assay and determination of nitric oxide production

J774A.1 and RAW 264.7 macrophages were cultured in supplemented RPMI-1640 medium (2 mM L-glutamine, 100 µg/mL streptomycin/penicillin, and 5% fetal bovine serum) at 37 °C in a 5% CO₂ atmosphere. Cytotoxicity was assessed using the 3-(4,5-dimethylthiazol-2-yl)-2,5-diphenyltetrazolium bromide assay (MTT assay) [17]. Macrophages were plated (2 × 10⁵ cells/mL) in 96-well plates and incubated for 48 h (37 °C, 5% CO₂) in the presence or absence of the ethanolic extract from EEB at different concentrations (100, 33.3, and 10 µg/mL). After MTT incubation, formazan crystals were dissolved and absorbance was read in a plate spectrophotometer (EZ Read 2000, Biochrom, Holliston, MA, USA) at 570 nm. Viability (%) was calculated as (X1/X2)*100, in which X1 and X2 are respectively the mean optical density (OD) (570 nm) for treated and untreated cells. A 70% cytotoxicity level was used, as determined by ISO 10993-5:2009 [18]. To determine nitric oxide (NO) production, the cells were cultured for 48 h in the presence or absence of EEB (100, 33.3, and 10 µg/mL) and stimulated with lipopolysaccharide (LPS) (1 µg/mL) and Interferon gamma (IFN-γ) 0.9 ng/mL. After incubation, the supernatants were collected for analysis of the NO concentration using the Griess method [19]. One hundred µL of supernatant was transferred to 96-well plates and an equal volume of Griess reagent (1% sulfanilamide, 0.1% N-(1-naphthyl)-ethylene diamine hydrochloride, and 2.5% H₃PO₄) was added. The concentration of NO was determined by comparison with a standard solution of sodium nitrite read in the EZ Read 2000 at 540 nm.

### Experimental model

Female BALB/c mice (6–8 weeks of age) were purchased from the Central Vivarium/UFOP and kept in ventilated cages. Filtered water and NUVILAB-CR1 feed were provided *ad libitum*. The experiment received approval from the Mucuri/UFVJM Animal Experimentation Ethics Committee under protocol number 01-2025 R and all procedures were carried out in accordance with the principles of the Brazilian Code for the Use of Laboratory Animals.

The mice were weighed and randomly assigned to nine different experimental groups (*n* = 5 animals/group): (1) negative control, (2) carrageenan + PBS, (3) carrageenan + dexamethasone, (4) EEB 100 mg/kg, (5) EEB 200 mg/kg, (6) EEB 100 mg/kg + LA meridians, (7) EEB 200 mg/kg + LA meridians, (8) LA meridians, and (9) local laser. At 0 h, the paws were measured with calipers (Mitutoyo, model 547-301B, Japan). Sixty minutes before the induction of edema, mice were treated or not treated with 100 µL of phosphate-buffered saline (PBS), EEB (100 or 200 mg/kg), or dexamethasone (0.5 mg/kg). Carrageenan (2.5%) [20] was dissolved in PBS and injected (20 µL) into the plantar pad of the left paw. PBS (20 µL) was injected into the right paw as control. One hour after injection, the groups of animals treated with 100µL of PBS or EEB (100 or 200 mg/kg) underwent laser acupuncture at acupoints ST36 (Zunsali) and SP6 (Sanynjiao) [21] at a wavelength of 808 nm (infrared) using the ECCO FIBRAS ALgaAs device (Brazil) (power: 120 mW; time: 25 s; energy density: 9.7 J/cm^2^; beam output area: 0.31 cm^2^; energy: 3 J; power density: 0.39 W/cm^2^) [22–24] and one group treated with PBS was submitted to local laser application of the plantar pad of left paw at the same wavelength using the same device and parameters.

During LA, the animals were restrained manually by a single trained operator without the use of sedation. Mild physical containment was applied to ensure the precise positioning of the laser pen while minimizing stress. The right and left paws were measured one, two, three, and four hours after the injection of carrageenan. Edema was calculated using the formula: ΔPaw thickness (mm) = T_carrageenan_−T_PBS_ (change in paw thickness (mm) = thickness (mm) of paw injected with carrageenan – thickness (mm) of paw injected with PBS). One group in which edema was not induced remained untreated and used as the negative control. After the measurement at the 4th hour, the mice were euthanized under intraperitoneal anesthesia (ketamine 100 mg/kg and xylazine 10 mg/kg). The left paw was removed and frozen at -80 °C for subsequent cytokine analysis.

### Maceration of soft tissue of paw

The plantar pad was excised, weighed, and macerated with 1 mL of 0.4 M NaCl (Isofar Indústria e Comércio), 0.05% Tween 20 (Merck & Co. Inc., Whitehouse Station, USA), 0.5% bovine serum albumin (BSA) (Sigma), 0.1 M phenyl-methyl-sulfonyl fluoride, 0.1 M benzethonium chloride (Sigma), 10 mM ethylenediaminetetraacetic acid (Sigma), and 20 pM aprotinin (Sigma). The macerates were homogenized and centrifuged at 10,000 rpm for 15 min at 4 °C [25]. The supernatants were collected and frozen at -80 °C for the cytokine assay.

### Concentrations of IL-1β and IL-6

The supernatants of the macerates were assayed for cytokines IL-1β and IL-6 (BD Biosciences Pharmingen, San Diego, CA, USA) using an enzyme-linked immunosorbent assay (ELISA), following the manufacturer’s protocol. Readings were performed in the EZ Read 2000 at 450 nm and concentrations were determined using standard curves [26].

### Statistical analysis

Statistical analyses were carried out with the aid of GraphPad Prism version 5.0. Data were expressed as mean ± standard error of the mean of at least three independent experiments. Significant differences between groups were determined using ANOVA, the Kruskal-Wallis test, or two-way ANOVA, when appropriate. Differences were considered significant when *p* < 0.05 [26].

## Results

### In vitro analysis of ethanolic extracts of Bauhinia monandra Kurz

**Fig. 1:**
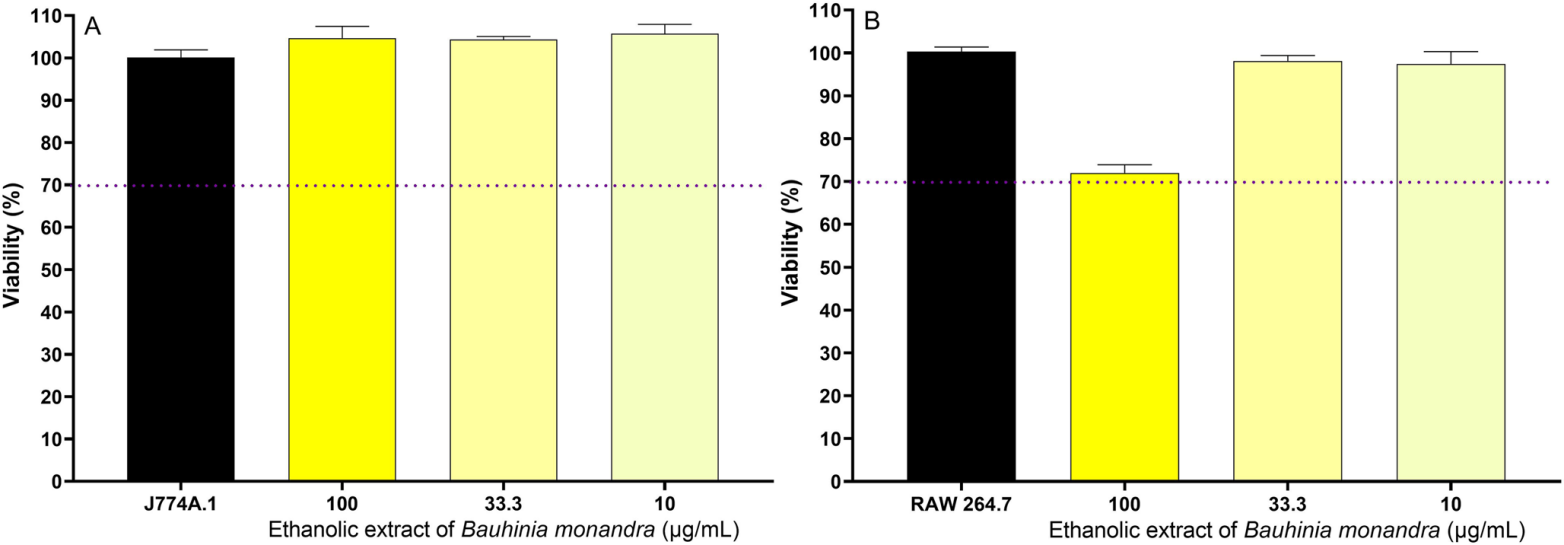
Effect of the ethanolic extract of Bauhinia monandra Kurz (EEB) on the cell viability of J774A.1 (A) and RAW 264.7 (B) murine macrophages after treatment with *Bauhinia monandra* Kurz ethanolic extract (EEB). Cells were incubated for 48 h with different concentrations of the extract (10–100 µg/mL). Each bar represents the mean ± SEM of three independent experiments. Dashed line = 70% threshold of cytotoxicity

Regarding the in vitro assays, the effects of the ethanolic extract of EEB on the viability of J774A.1 (A) and RAW 264.7 (B) macrophages were evaluated after 48 h of incubation. Cell viability was assessed using the MTT assay, where the dashed line represents the 70% viability threshold established by ISO 10993-5:2009 [18] for cytotoxicity classification, as shown in Fig. 1.

**Fig. 2:**
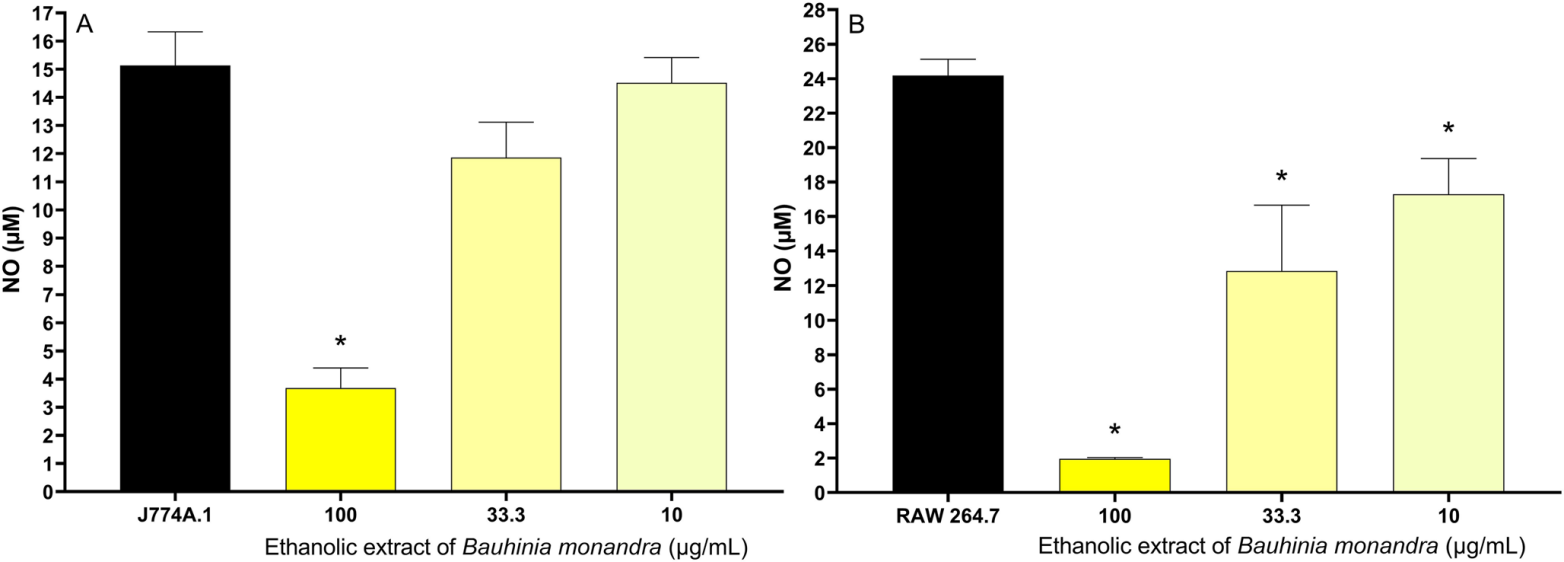
Effect of the ethanolic extract of Bauhinia monandra Kurz (EEB) on nitric oxide (NO) production in J774A.1 (A) and RAW 264.7 (B) murine macrophages stimulated with LPS (Lipopolysaccharide) + IFN-γ and treated with *Bauhinia monandra* ethanolic extract (EEB) at different concentrations (10–100 µg/mL). Each bar represents the mean ± SEM of three independent experiments. * *p* < 0.05 when compared to untreated cells (J774A.1 or RAW 264.7)

The results are expressed as mean ± SEM, and comparisons with the untreated control are shown in Fig. 1. NO production was assessed in LPS/IFN-γ-stimulated J774A.1 and RAW 264.7 macrophages after treatment with EEB. EEB reduced NO production compared with the stimulated control group (EEB100 vs. J774A.1 *p* = 0.0091; EEB100 vs. J774A.1 *p* = 0.0091; EEB33.3 vs. J774A.1 *p* = 0.031; EEB10 vs. J774A.1 *p* = 0.039), as indicated by the statistical comparisons shown in Fig. 2. The results are expressed as mean ± SEM, and the significance levels are specified in the figure legend.

### Phytochemical profile of ethanolic extract of Bauhinia monandra Kurz

The HPLC-MS analysis of EEB revealed the predominance of phenolic compounds, especially flavonoids and phenolic acids. The chromatogram (Appendix − 1 A) illustrates the retention profile of the metabolites over 60 min of chromatographic analysis by LC-MS, reflecting both differences in polarity and interactions with the stationary phase. Most compounds eluted after the first 15 min, indicating the predominance of metabolites of intermediate polarity. However, the major metabolites were detected between 35 and 50 min, suggesting the presence of medium to low polarity substances, which tend to have higher chromatographic retention. Concomitantly, the mass spectrum (Appendix − 1B) showed a significant abundance of ions in the range of 100 to 500 Da, which is consistent with the presence of low to medium molecular weight metabolites, such as phenolic acids, simple phenols, and flavonoids. The detection of compounds of higher molecular weight suggests the occurrence of triterpene saponins and some glycosylated flavonoids, which have more complex, highly oxygenated structures (Table 1). The analysis of the van Krevelen diagram (Appendix − 1 C) generated from the O/C and H/C ratios (Xcalibur software) revealed the formation of characteristic clusters: regions with higher O/C ratios and intermediate values of H/C are typical of oxidized phenolic derivatives; areas with low O/C and H/C indicate more aromatic structures, compatible with flavonoid aglycones; while points located at high O/C values and low H/C suggest carbohydrate-rich molecules such as glycosylated flavonoids and saponins.

**Table 1:**
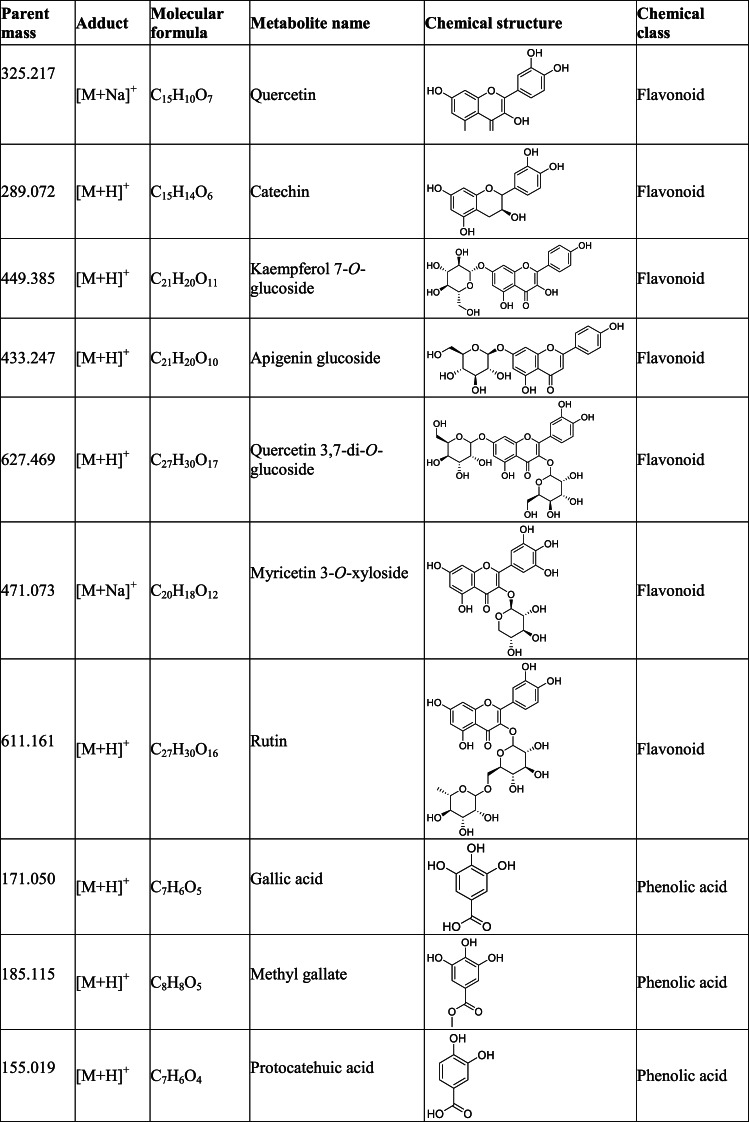
Phenolic compounds putatively identified in Bauhinia monandra extracts by high-resolution mass spectrometry. The table includes the observed parent ion mass, detected adducts, molecular formulas, metabolite annotations, chemical structures, and corresponding chemical classes

### Effect of ethanolic extract of Bauhinia monandra combined with laser acupuncture on carrageenan-induced paw edema

**Fig. 3:**
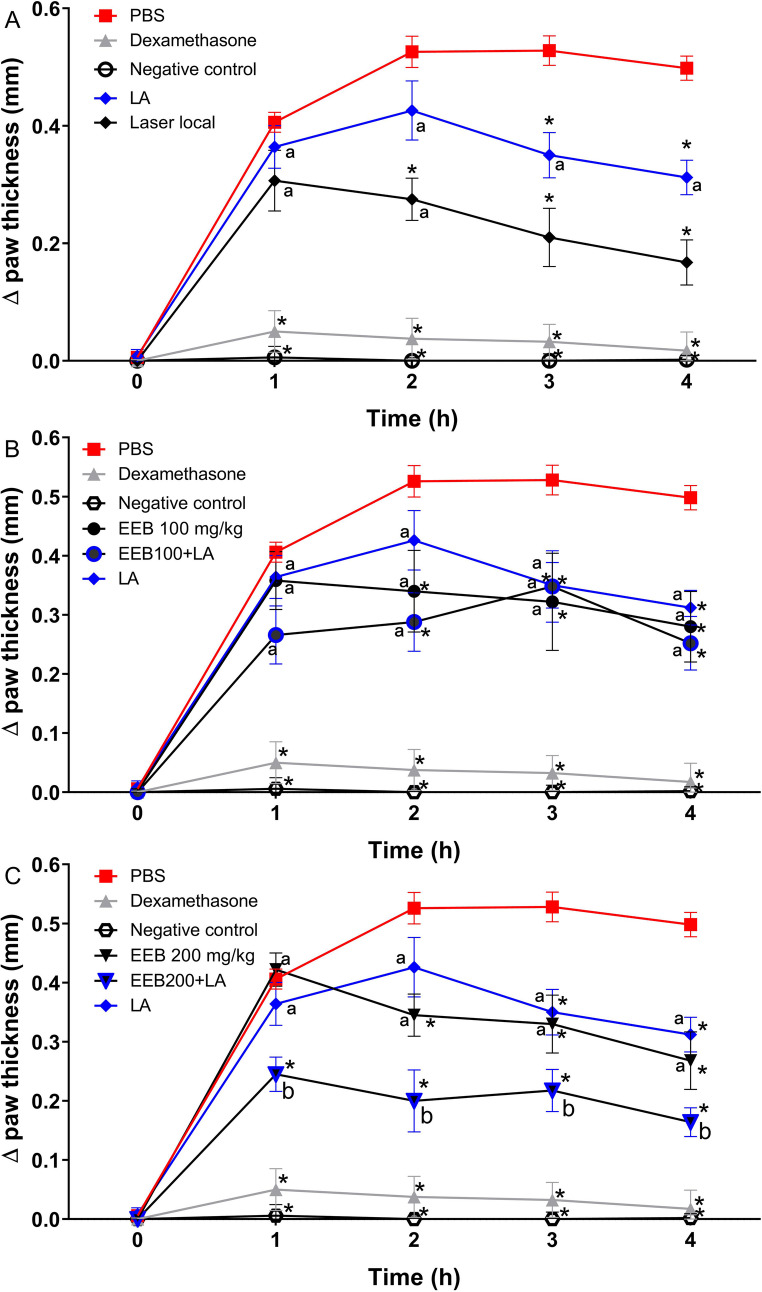
Effect of *Bauhinia monandra* Kurz ethanolic extract (EEB), laser acupuncture (LA), and local laser application on carrageenan-induced paw edema in BALB/c mice. Edema was evaluated at 0, 1, 2, 3, and 4 h after induction. Treatments included phosphate-buffered saline (PBS), EEB (100 or 200 mg/kg), LA at acupoints ST36 and SP6, local laser application in the left paw, EEB + LA combination, and dexamethasone. Data represent mean ± SEM from three independent experiments. *p < 0.05 compared to PBS (induced control group). ^a^*p*<0.05 when compared to the dexamethasone. ^b^*p*<0.05 when compared to the EEB 200 mg/kg. Negative control was non-induced and untreated mice

Figure 3 illustrates carrageenan-induced paw edema and the effects of EEB treatment. Carrageenan produced prominent edema in the left paw, peaking at 0.528 ± 0.025 mm at 3 h (Fig. 3A–C). Laser acupuncture (LA) reduced edema to a similar extent as local laser application (Fig. 3A). The results of local laser did not differ significantly from those achieved with dexamethasone at 3 and 4 h (Fig. 3A). Both EEB concentrations (100 or 200 mg/kg) reduced edema from 2 h onward (Fig. 3B). At 100 mg/kg, the association with LA did not produce a statistically significant additional reduction compared with EEB100 alone. In contrast, only EEB at 200 mg/kg combined with LA was statistically indistinguishable from dexamethasone (Fig. 3B, C) and showed lower edema when compared to EEB at 200 mg/kg alone (*p* = 0.0437 at 4th hour).

### IL-1β and IL-6 modulation in paw tissue

**Fig. 4:**
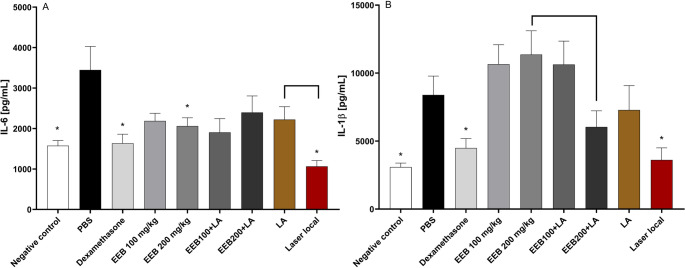
Effect of *Bauhinia monandra* Kurz ethanolic extract (EEB), laser acupuncture (LA), and local laser application on IL-6 (A) and IL-1β (B) levels in paw tissue after carrageenan-induced edema. Treatments included phosphate-buffered saline (PBS), EEB (100 or 200 mg/kg), LA at acupoints ST36 and SP6, local laser application in the left paw, EEB + LA combination, and dexamethasone. Data represent mean ± SEM from three independent experiments. *p < 0.05 compared to PBS (induced control group). Bracket *p* < 0.05 between groups. Negative control was non-induced and untreated mice

In Fig. 4, when cytokine production was assessed, both dexamethasone and local laser reduced IL-1β and IL-6 levels, whereas EEB 200 mg/kg reduced only IL-6 levels when compared to carrageenan + PBS. However, EEB at 200 mg/kg combined with LA showed lower IL-1β level when compared directly to EEB at 200 mg/kg alone. Carrageenan induced an increase in both IL-1β (8392.0 ± 1386.0 pg/mL vs. 3083.0 ± 297.0 pg/mL in negative control) and IL-6 (3447.0 ± 583.2 pg/mL vs. 1576.0 ± 128.5 pg/mL in negative control). Moreover, local laser led to a greater reduction in IL-6 compared to LA. Demais tratamentos não modularam a produção de IL-6 ou IL-1 em relação ao grupo carragenina, ou as suas contrapartes.

## Discussion

The present study demonstrated that the ethanolic extract of EEB exerts a significant anti-inflammatory effect in in vitro and in vivo models, and that the combination with laser acupuncture (LA) enhances these effects, resulting in responses comparable to the reference pharmacological treatment in the carrageenan-induced model of paw edema. These findings demonstrate the potential of combining therapies based on natural products and photobiomodulation for the control of inflammation.

Phenolic compounds constituted one of the major metabolite classes identified in *Bauhinia monandra*. The detected phenolic profile was predominantly represented by flavonoids, including quercetin, catechin, kaempferol 7-*O*-glucoside, apigenin glucoside, quercetin 3,7-di-*O*-glucoside, myricetin 3-*O*-xyloside, and rutin, together with simple phenolic acids such as gallic acid, methyl gallate, and protocatechuic acid. These compounds are widely recognized for their antioxidant and anti-inflammatory, contributing significantly to the biological potential of *Bauhinia* species. The occurrence of both aglycone and glycosylated flavonoids suggests a diversified phenolic metabolism, while the presence of gallic acid derivatives highlights the contribution of hydroxybenzoic acid pathways to the chemical composition of the species. These phenolic compounds have been widely reported in the genus *Bauhinia*, including in *B. monandra*, supporting the reliability of their annotation in the present study [11, 27].

In the in vitro assays, EEB exhibited no cytotoxicity in murine macrophages and promoted the significant inhibition of LPS-induced NO production. The inhibition of NO production is in agreement with previous reports describing the ability of flavonoids to modulate iNOS expression in activated macrophages [28–30].

In the in vivo model, carrageenan-induced paw edema was employed for its robustness in the assessment of anti-inflammatory agents, characterized by the biphasic release of vasoactive mediators that culminates in the high production and release of pro-inflammatory cytokines [5]. The findings of the present study are in line with recent evidence in the literature that highlights the high potential effect of photobiomodulation combined with plant extracts [4, 31, 32]. Indeed, previous studies have demonstrated the anti-inflammatory potential of species of the genus *Bauhinia*, which is often associated with the presence of bioactive phenolic compounds [9, 12].

The application with LA at acupoints ST36 (Zusanli) and SP6 (Sanyinjiao) also resulted in significant reductions in edema as well as the pro-inflammatory cytokines IL-1β and IL-6. These results are in line with data described in previous studies reporting that the stimulation of these points can modulate the systemic inflammatory response, possibly through neuroimmune pathways and the regulation of intracellular mediators, such as NF-κB [14–16, 32]. Photobiomodulation has been associated with increased mitochondrial activity and adenosine triphosphate (ATP) production, thus favoring tissue repair processes [14, 15].

The comparison of LA by meridians and local LA revealed that both approaches were effective at reducing edema, especially local application. This finding suggests that distinct mechanisms may be involved, including direct local effects on inflamed tissue and systemic effects. Esse achado sugere que a ação direta da fotobiomodulação sobre o tecido inflamado pode desempenhar um papel relevante na modulação de uma resposta inflamatória. Embora a estimulação dos acupontos seja frequentemente associada à ativação de vias neuroimunes sistêmicas, a aplicação local pode possibilitar uma entrega direta da energia luminosa ao foco inflamatório, o que poderia potencializar efeitos celulares e metabólicos no tecido-alvo, conforme os resultados observados.

The combination of EEB and LA promoted a more pronounced anti-inflammatory effect than the individual treatments, indicating a potential synergic interaction. Similar results have been described in previous studies that investigated the combination of plant extracts and photobiomodulation using the same experimental model, with significant reductions in edema and inflammatory cytokines [4, 33, 34]. Further studies are needed to clarify the molecular mechanisms involved in this interaction and assess the effectiveness of combined therapy in models of chronic inflammation. 

### Limitations

Although promising, these results present limitations inherent to experimental design. First, the exclusive use of the acute carrageenan-induced paw edema model, restricted to a 4-hour post-induction evaluation, precludes the extrapolation of the combined therapy's effects to chronic conditions. Phytochemically, while analysis identified the major metabolite classes, it did not isolate or individually evaluate the bioactive molecules; furthermore, a single batch of extract was used, preventing the assessment of chemical variability between different preparations. Regarding laser acupuncture (LA), the protocol employed fixed dosimetric parameters, making it impossible to evaluate the influence of varying wavelengths, energy densities, or application times. The sample size (𝑛 = 5 animals/group), though approved by the institutional ethics committee (CEUA), may have limited the statistical power to detect differences in specific pair wise comparisons, such as between EEB100 and EEB100+LA; therefore, the lack of significance in these specific cohorts does not definitively rule out an additive effect of LA. Additionally, the manual restraint of animals without sedation may induce stress responses capable of modulating neuroimmune and inflammatory parameters. Although minimized by standardizing the operator and protocol across all groups, this factor cannot be completely discounted. Finally, as a pre-clinical study, future investigations are essential to elucidate the molecular mechanisms involved, confirm reproducibility in chronic models, and evaluate the translational potential of this approach. 

## Conclusion

The ethanolic extract of *Bauhinia monandra* Kurz exhibited significant anti-inflammatory activity, as demonstrated by the inhibition of nitric oxide production in macrophages as well as the reductions in paw edema and the pro-inflammatory cytokines IL-1$\beta$ and IL-6 in the carrageenan-induced model. Moreover, LA – whether applied to ST36 and SP6 acupoints or locally – was shown to be effective in controlling the inflammatory response. Notably, the combination of the extract of *B. monandra* Kurz and laser acupuncture enhanced these effects, promoting responses comparable to the reference pharmacological treatment, with no evidence of cytotoxicity. The combination of these approaches leads to an enhanced anti-inflammatory effect, possibly due to the interaction between the cellular modulation induced by photobiomodulation and the bioactive action of the phytochemicals. However, given that the present study was conducted in a pre-clinical model of acute inflammation, further studies are warranted to elucidate the underlying mechanisms of the observed effects and to determine the applicability of this approach in more complex experimental models.

## Supplementary Information

Below is the link to the electronic supplementary material.

Supplementary Material 1

## Data Availability

All data supporting the findings of this study are available within the paper.
