# Supplementary material for: Combination of Bauhinia monandra extract and laser acupuncture reduces carrageenan-induced paw edema
Source: Lasers Med Sci. 2026 Aug 10;41(1):182. doi: 10.1007/s10103-026-04986-2 (PMC13453983; doi:10.1007/s10103-026-04986-2)
Supplement: Supplementary file 1 [file 10103_2026_4986_MOESM1_ESM.docx]

**Appendix:** **(A)** LC-MS chromatogram obtained using an HPLC-UV 1220 Infinity II system (Agilent Technologies) coupled to a Q-Exactive Hybrid Quadrupole-Orbitrap high-resolution mass spectrometer (Thermo Scientific) operating with an electrospray ionization source in positive mode.

**(B)** Mass spectrum acquired in the $m/z$ 100–1000 range, highlighting the ions detected in the analyzed extract.

**(C)** Van Krevelen diagram corresponding to the molecular formulas assigned by Xcalibur software, representing the distribution of compounds based on H/C and O/C atomic ratios.
